# The Placental Epigenome as a Molecular Link Between Prenatal Exposures and Fetal Health Outcomes Through the DOHaD Hypothesis

**DOI:** 10.1007/s40572-022-00354-8

**Published:** 2022-04-29

**Authors:** Samantha Lapehn, Alison G. Paquette

**Affiliations:** 1 Center for Developmental Biology and Regenerative Medicine, Seattle Children’s Research Institute, 1900 9th Avenue, Seattle, WA 98101 USA; 2 Department of Pediatrics, University of Washington, Seattle, WA USA

**Keywords:** Placenta, Epigenomics, DOHaD, Exposure, Methylation, Transcriptome

## Abstract

**Purpose of Review:**

The developmental origins of health and disease (DOHaD) hypothesis posits that the perinatal environment can impact fetal and later life health. The placenta is uniquely situated to assess prenatal exposures in the context of DOHaD because it is an essential ephemeral fetal organ that manages the transport of oxygen, nutrients, waste, and endocrine signals between the mother and fetus. The purpose of this review is to summarize recent studies that evaluated the DOHaD hypothesis in human placentas using epigenomics, including DNA methylation and transcriptomic studies of mRNA, lncRNA, and microRNAs.

**Recent Findings:**

Between 2016 and 2021, 28 articles evaluated associations between prenatal exposures and placental epigenomics across broad exposure categories including maternal smoking, psychosocial stressors, chemicals, air pollution, and metals. Sixteen of these studies connected exposures to health outcome such as birth weight, fetal growth, or infant neurobehavior through mediation analysis, identification of shared associations between exposure and outcome, or network analysis. These aspects of infant and childhood health serve as a foundation for future studies that aim to use placental epigenetics to understand relationships between the prenatal environment and perinatal complications (such as preterm birth or fetal growth restriction) or later life childhood health.

**Summary:**

Placental DNA methylation and RNA expression have been linked to numerous prenatal exposures, such as PM2.5 air pollution, metals, and maternal smoking, as well as infant and childhood health outcomes, including fetal growth and birth weight. Placental epigenomics provides a unique opportunity to expand the DOHaD premise, particularly if research applies novel methodologies such as multi-omics analysis, sequencing of non-coding RNAs, mixtures analysis, and assessment of health outcomes beyond early childhood.

## Introduction

### The Placenta and the DOHaD Hypothesis

The placenta is an important regulator of the fetal environment with roles in nutrient transport, oxygen and waste exchange, and endocrine signaling [1]. The developmental origins of health and disease (DOHaD) hypothesis states that environmental exposures spanning the perinatal period through birth can impact disease outcomes in later life [2, 3]. The placenta is an ephemeral fetal organ that embeds in the maternal decidua. It is uniquely situated to assess the DOHaD hypothesis due to its position as an interface exchanging maternal physiological and environmental cues with the developing fetus [4]. The placenta attaches to the fetus via the umbilical cord which branches into villous trees that exchange oxygen, nutrients, waste, and endocrine signals between the fetus and the placenta [5]. Placental omics data, including transcriptomics and epigenomics, have been linked to a number of maternal and postnatal health outcomes as well as environmental exposures [6]. Since the placenta is an essential, functional tissue of gestation that is amenable to collection after delivery, it is more ideal for these assessments than proxy measures such as saliva because placental omics may directly reflect perturbations in placental function that can impact the fetus. While the DOHaD hypothesis is now over 30 years old, omics technology did not become widely available until the last decade, meaning that application of omics approaches to assess the DOHaD premise in the placenta is only just beginning [7].

### Epigenetics

Epigenetics is the study of how transient marks to the human genome affect gene expression potential. Early epigenetic research utilized candidate gene approaches to investigate epigenetic regulation of selected genes believed to be associated with a condition of interest. The advent of omics technologies moved the field of epigenetics to expand into hypothesis-generating designs which are broadly referred to as epigenomics [8]. There are several forms of epigenetic mechanisms that can affect downstream gene expression. Histone modifications are epigenetic marks on the proteins (histones) that regulate chromatin compaction and DNA accessibility. Acetyl and methyl modifications are the two most common types of histone modifications, and they differ in their effect on gene expression (activating or repressing) based on the number and location of marks on the histone complex [9]. DNA methylation plays an essential role in mammalian development as a function to reprogram the genome at fertilization and germline cell specification [10]. DNA methylation is the direct addition of a methyl group to a DNA base, most frequently occurring on cytosines that are adjacent to guanine residues. When this occurs within the promoter region of a gene, it is traditionally associated with gene silencing through recruitment of repressors and inhibition of transcription factors [11]. However, in some contexts, DNA methylation is shown to activate expression [12]. Hydroxymethylation of these cytosine residues is also believed to play a role as an epigenetic intermediate that may affect gene expression potential [13]. Genome-wide DNA methylation can be easily assessed in large-scale human populations through DNA methylation arrays; however, there is not yet a comparable technology for assessing histone modifications in large epidemiologic studies due to issues related to antibody reproducibility and tissue quantity and quality required for processing [14].

Non-protein coding RNA molecules, such as long non-coding RNAs (lncRNAs) and microRNAs (miRNAs), are epigenetic regulators of mRNA expression. MicroRNAs are a subtype of small, non-coding RNAs which bind to target mRNAs and regulate expression by causing destabilization, preventing translational initiation, or inducing de-adenylation and decay [15]. MicroRNAs are expressed within the placenta and are involved in fetal and maternal signaling through secretion into maternal circulation within extracellular vesicles known as exosomes [16]. Identification of placenta-specific microRNAs secreted into the maternal blood from exosomes is an area of growing interest as a non-invasive biomarker for pregnancy health [17].

lncRNAs are less well studied, but can regulate gene expression by binding to promoter regions of target genes or remodeling chromatin [18]. Post-transcriptional editing of RNA molecules by RNA editing proteins (including readers, writers, and erasers) can affect RNA splicing resulting in splice variants, changes to gene expression, and changes in binding capability of microRNAs, all resulting in transcriptomic alterations [19]. Although gene expression itself is not considered an epigenomic regulator, mRNA expression levels are a reflection of the direct effects of upstream epigenetic modification and will be included in this review as it is an important component of multi-epigenomics analyses. Most of these epigenetic methods of gene regulation have been studied in the placenta; however, their application to specific prenatal exposures and postnatal health outcomes is still a growing area of research.

**Fig. 1:**
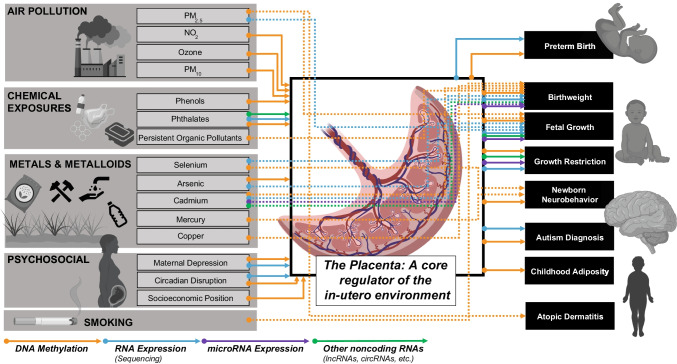
Associations and connections between placental exposures and birth/early childhood health outcomes measured through placental epigenomics

There is a large body of research involving placental epigenetics and the DOHaD hypothesis, which has been extensively reviewed [4, 20, 21]. Many of these previous studies were designed to interrogate specific candidate genes or genomic regions, based on prior evidence or hypothesis. As large-scale omic studies have become more feasible, this review will focus on transcriptomic and epigenomic changes assessed in human populations specifically using omics-based approaches that have been published between 2016 and 2021. Human studies evaluating exposures and/or postnatal health outcomes were included when they used DNA methylation arrays, RNA sequencing, microarrays, or panel studies of microRNAs and lncRNAs to evaluate differences within placental tissue (Table 1). We excluded studies involving nutritional and supplement exposures, maternal disease, or recreational or pharmaceutical drug exposures. Additionally, studies assessing the effect of maternal or fetal genetic variation on placental epigenomics were also excluded. Studies with no significant findings have also been excluded. The aim of this review is to summarize the state of environmental epigenomics research in the placenta and identify gaps in knowledge for future research (Fig. 1).

**Table 1:** Recent manuscripts categorized by environmental exposure (*no shared associations with exposure and outcome identified)

Exposure		Omic data analyzed	Cohort	*N*	Outcome	Analysis strategy	Reference
Air pollution	PM_2.5_	mRNA expression	RICHS	471	Fetal growth	Network	Deyssenroth 2021 [22]
		DNA methylation	SMCP	287	Fetal growth	Mediation	Zhao 2021 [23]
			COCOA	922	Atopic dermatitis	Network	Yang 2020 [24]
	PM_10_	DNA methylation	EDEN	668	-	-	Abraham 2018 [25]
	NO_2_	DNA methylation	EARLI	133			Ladd-Acosta 2019 [26]
			EDEN	668			Abraham 2018 [25]
	Ozone	DNA methylation	EARLI	133			Ladd-Acosta 2019 [26]
Chemical	Phenols	DNA methylation	EDEN	202	-	-	Jedynak 2021 [27]
			SMBC	6			Song 2021 [28]
	Phthalates	lncRNA expression	-	20			Machtinger 2018 [29]
		mRNA & lncRNA expression	CANDLE	760			Paquette 2021 [30]
		DNA methylation, mRNA expression	-	34			Grindler 2018 [31]
	POPs	DNA methylation	NICHD-FGS	260	Birth weight	Mediation	Ouidir 2020 [32]
Metals	Selenium	DNA methylation	NHBCS, RICHS	484	NNNS score	Overlap	Tian 2020 [33]
		mRNA expression	RICHS	173	Fetal growth	Overlap	Everson 2017 [34]
	Arsenic	mRNA expression	NHBCS	48	Birth weight	Network	Winterbottom 2019a [35]
				311	-	-	Winterbottom 2019b [36]
		DNA methylation		343			Green 2016 [37]
	Cadmium	mRNA expression	RICHS	173	Fetal growth	Overlap	Everson 2017 [34]
		lncRNA expression		259	Birth weight	Overlap	Hussey 2020 [38]
		DNA methylation, mRNA expression	RICHS, NHBCS	484		Overlap	Everson 2018 [39]
		microRNA expression	-	36		Overlap	Clark 2021 [40]
	Copper	DNA methylation	RICHS, NHBCS	447		Network	Kennedy 2020 [41]
	Mercury		RICHS	151	NNNS score	Overlap	Maccani 2015 [42]
	Metal mixture		RICHS	195	Fetal growth	Overlap	Deyssenroth 2018 [43]
Psychosocial	Maternal depression	mRNA expression	RICHS	581	NNNS score*	Overlap	Litzky 2018 [44]
		DNA methylation	FinnBrain	92	-	-	Lund 2021 [45]
	Circadian disruption	DNA methylation, mRNA expression	RICHS	237			Clarkson-Townsend 2019 [46]
	SES	DNA methylation	ELGAN	426			Santos 2019 [47]
Smoking	Maternal smoking	DNA methylation	INMA	179	Birth weight	Mediation	Morales 2016 [48]
			Gen3G	441	Birth weight	Mediation	Cardenas 2019 [49]
			PACE	1700	Birth weight, fetal growth	Overlap	Everson 2021 [50]

## Placental Epigenomics and Prenatal Exposures

### Maternal Smoking

Within the last 5 years, three epigenomic analyses have assessed the relationship between placental DNA methylation and self-reported maternal smoking. The Genetics of Glucose Regulation in Gestation and Growth (Gen3G) cohort assessed DNA methylation in 441 maternal-infant dyads which revealed 71 differentially methylated CpG sites (*p* < 6.94 × 10^−8^) associated with maternal smoking [49]. Seven of these CpG sites were significant mediators of the relationship between maternal smoking and infant birth weight (FDR < 0.05). Placental DNA methylation was also studied in the INfancia y Medio Ambiente (INMA) cohort from Spain (*N* = 179), which identified 50 CpG sites with differential methylation between smoking and non-smoking mothers [48]. The 14 CpGs with > 5% methylation differences based on smoking status were used in a mediation analysis which identified 2 CpGs associated with differences in infant birth weight [48]. Maternal smoking was also assessed in a meta-analysis of seven individual studies within the Pregnancy and Childhood Epigenetics (PACE) consortium (*N* = 1700) which identified 443 CpG sites significantly associated with maternal smoking. A fraction of these CpGs were also associated with gestational age (121 CpGs), preterm birth (44 CpGs), birth weight (25 CpGs), birth length (11 CpGs), and head circumference (2 CpGs) [50]. Thirty-four CpGs were significantly associated with maternal smoking by both Morales et al. and Everson et al. [48, 50]. Cardenas et al. did not include a list of the 89 CpGs associated with maternal smoking [49]. Two CpGs (cg27402634-*LINC00086*, *LEKR1* and cg20340720-*WBP1L*) were associated with both infant birth weight and maternal smoking in Morales et al. and Everson et al. [48, 50]. Pathway analysis of genes mapped to CpGs associated with maternal smoking revealed four significant pathways that were shared in these two studies including G Protein Signaling Pathways, Myometrial Relaxation and Contraction Pathways, Pathways in Cancer (*Homo sapiens*), and Signaling by FGFR in disease [48, 50]. Overall, these studies reveal that placental DNA methylation may partially mediate the relationship between maternal smoking and fetal growth or birth weight. Future studies of maternal smoking should evaluate the effect on the placental transcriptome as well as infant and child health outcomes such as growth, adiposity, and neurodevelopment.

### Chemicals

Pregnant individuals are exposed to a broad variety of manufactured chemical substances through consumer products, within the workplace, or in industrial settings. Phthalates are a type of plasticizer that can act as an endocrine-disrupting chemical and are associated with adverse birth outcomes. Phthalates have been studied in first trimester maternal urine and placentas (*N* = 34) with respect to DNA methylation (*N* = 16) and gene expression (*N* = 14) [31]. There were 163 differentially expressed genes (*p* < 0.005) and 2214 differentially methylated single CpG sites (*p* < 0.005) identified between women in the high vs. low total maternal phthalate groups [31]. Machtinger et al. examined the relationship between concentration of 15 phthalate metabolites measured in maternal urine at delivery and expression of 87 candidate lncRNAs from 10 twin pregnancies (*N* = 20) [29]. There were 35 associations (*p* < 0.05) between lncRNA expression and 10 individual phthalate metabolites, with only LOC91450 remaining significant after Bonferroni correction [29]. Maternal urinary concentrations of 21 phthalate metabolites in the 2nd and 3rd trimester of pregnancy and associations between the placental transcriptome have been studied in the Conditions Affecting Neurocognitive Development and Learning in Early Childhood (CANDLE) study (*N* = 760) [30]. Four phthalate metabolites were significantly associated with 38 genes, including four lncRNAs in either the 2nd or 3rd trimester, and several metabolites exhibited sex-specific relationships with placental gene expression [30]. Of particular note, Machtinger et al. and Paquette et al. both identified significant associations between phthalate metabolites and the lncRNA *NEAT1* with MMP (Machtinger) and MCIOP, MECCP, and MEOHP (Paquette), respectively [29, 30]. None of the differentially methylated genes identified by Grindler et al. were differentially expressed in the other studies [31]. Phthalates are associated with a number of prenatal outcomes such as preterm birth, so future directions may include using omics data to link exposures and birth outcomes as well as analyses that integrate more than one omics measure.

Phenol chemicals are industrial precursor chemicals with endocrine-disrupting effects that are used in the production of a diverse array of consumer products including polycarbonates, ultraviolet filters, biocides, and preservatives. Placental methylation of 282 CpGs within 208 genes (*p* < 0.05) were significantly differentially methylated (127 hypomethylated, 155 hypermethylated) between participants with BPA quantifiable in urine at 12–16 weeks compared to samples without (*N* = 3/group) within the Shanghai-Minhang Birth Cohort study [28]. Maternal urinary concentrations of 9 phenols and genome-wide placental DNA methylation were also evaluated in the French Etude des D’eterminants pr’e et postnatals du d’eveloppement et de la sant’e de l’Enfant (EDEN) cohort (*N* = 202) [27]. 596 CpGs (FDR < 0.05) were associated with urinary phenol concentrations which encompassed 406 known genes and 162 intergenic regions, with most of the significant findings involving triclosan [27]. There are many gaps in knowledge involving phenol exposure as it relates to the DOHaD premise, including connecting phenol exposures to birth and later childhood health outcomes, transcriptomic analysis of mRNAs and non-coding RNAs, and evaluating lesser-studied phenols.

Persistent organic pollutants (POPs) encompass a broad range of environmental pollutants including contaminants such as polyfluoroalkyl substances (PFAS) that are of emerging recent interest due to their ability to bioaccumulate, resulting in widespread exposure across the globe. Maternal plasma concentrations of 13 POP chemicals in early pregnancy were evaluated for associations with placental DNA methylation and gene expression in 260 dyads from the National Institute of Child and Human Development (NICHD) Fetal Growth Studies-Singleton cohort [32]. Methylation of 214 CpG sites from 205 genes was associated (FDR < 0.05) with maternal plasma POP concentrations [32]. These genes were members of pathways related to embryonic cell differentiation and brain size and morphology. Forty-four CpG sites that were significantly associated with POPs were also associated with least one measure of neonatal anthropometry, and 7 CpGs were significant mediators of the relationships between PBDE 47, a POP flame retardant, and either birth weight or head circumference [32]. Future directions for POP research include evaluating non-coding RNA expression and studying a wider variety of POPs individually and as mixtures. Overall, we identified only six studies involving chemical exposures with placental omics data in the last 5 years, despite chemicals being one of the largest human exposure categories that continues to rapidly evolve as older chemicals are replaced by new alternatives. In addition to keeping pace with new chemical introductions, chemical exposure research should also prioritize mixture analysis to be more reflective of actual human expsoures [51]. However, strategies to investigate chemical exposures through mixture analysis are not yet developed for both high dimensional exposure and omics data, and data reductionality techniques may be required.

### Metals

Metals and metalloids are a well-studied environmental exposure. While some metals like copper (Cu) and iron (Fe) are essential metals, others such as cadmium (Cd), lead (Pb), and mercury (Hg) are considered toxic, even at low concentrations [52]. Metalloids such as selenium (Se) and arsenic (As) have properties that fall between metals and other elements and similar to metals can be essential or toxic to humans [53]. Selenium is an essential metalloid element that humans are primarily exposed to through diet or occupational hazards. Se is an essential element and deficiencies have been linked to numerous adverse health outcomes including preterm birth and infant neurobehavior [33, 54]. In both the New Hampshire Birth Cohort Study (NHBCS, *N* = 343) and Rhode Island Child Health Study (RICHS) (*N* = 141), 8 differentially methylated CpG sites (FDR < 0.05) were associated with placental Se concentration. Increased methylation of *GFI1* was positively associated with Se concentrations and was associated with reduced odds of newborn muscle hypertonicity (OR = 0.85, *p* < 0.003) [33].

Cd is a toxic, heavy metal that is used in mining, industrial processes, fertilizers, and combustion processes. In the RICHS cohort, intrauterine growth restriction (IUGR) and small for gestational age (SGA) pregnancies had higher median Cd concentrations and lower median Se concentrations in maternal toenail samples collected postpartum representing long-term exposure (*N* = 173) [34]. A subset of genes involved TNF signaling (21 genes) and steroidogenesis (8 genes) that were previously associated with fetal growth and were also significantly associated with toenail Cd and Se concentrations as well as birth size [34]. An EWAS study of the combined RICHS and NHBCS cohorts (*N* = 484) revealed two differentially methylated CpGs (FDR adjusted *p* < 0.05) and 17 suggestively significant CpGs (unadjusted *p* < 0.00001) [39] associated with placental Cd concentrations. Of these 17 CpG sites associated with Cd, 9 were associated with 6 genes with altered gene expression in the RICHS cohort. Three genes (*TNFAIP2*, *ACOT7*, and *RORA*) were also associated with birth weight outcomes [39]. Birth weight was associated with expression of 46 lncRNAs in the RICHS cohort (*N* = 259), and 4 of these lncRNAs were also associated with placental Cd concentrations, suggesting that disruption of lncRNA expression may be part of Cd’s mechanism of reproductive toxicity [38].

Arsenic (As) is a metalloid contaminant of air, water, and soil that humans are primarily exposed to through water and diet. 164 CpG sites (FDR < 0.05) were differentially methylated with placental As concentration in the NHBCS cohort (*N* = 343), with the majority of CpGs demonstrating hypomethylation in association with increased arsenic [37]. A total of 606 differentially expressed genes (DEGs) were associated with placental arsenic concentration (FDR < 0.05) in male placentas, but no significant differences were observed in female placentas in the NHBCS (*N* = 48) [35]. In the same study, there were 103 gene sets in females and 100 in males that were associated with both birth weight and As exposure [35]. Placental expression of a panel of 138 epigenetic protein genes was also studied in relation to As exposure in mother infant pairs from the NHBCS cohort (*N* = 311), whose home drinking water was from private wells [36]. Twenty-seven of these genes were associated with maternal urinary As exposure during the 2nd trimester (*p* < 0.05) [36].

Mercury (Hg) is a liquid metal that humans are primarily exposed to through fish consumption, occupational exposures, or old dental fillings. Mercury exposure quantified in infant toenail samples was associated with both infant neurobehavior and placental DNA methylation within 339 CpG sites in the RICHS cohort (*N* = 151) [42]. Ten of the differentially methylated CpG sites were located within genes that were associated with a specific subset of infants with a higher risk neurobehavior profile than the rest of the infants in the cohort (*p* < 0.01) [42]. Copper (Cu) is an essential metallic trace nutrient that in high doses such as experienced in mining can cause human toxicity. Kennedy et al. identified 2 CpG sites with differential methylation in association with placental copper concentrations that were significant after Bonferroni correction (*p* < 1 × 10^−7^), in the RICHS (*N* = 141) cohort [41]. Four differentially methylated regions (*p* < 0.05) were enriched for zinc finger proteins, including *ZNF197*, which was co-expressed with 138 transcripts in RICHS that were associated with infant birth weight [41].

Simultaneous analysis of multiple compounds as a mixture is a growing area of research interest to better approximate real-life exposure scenarios. Only one study evaluated metals as a mixture through weighted quantile sum regression to derive metal mixture indices. Metal indexes derived from 16 trace metals quantified from postpartum toenail clippings were investigated in the context of SGA status and placental gene co-expression in the RICHS cohort (*N* = 195) [43]. The most significant finding was that a multi-metal index, dominated by As (44.4%) and Cd (17.8%), was significantly associated with both SGA status (OR = 2.37), and placental expression of genes involved in metabolic hormone secretion [43]. Overall, metals are one of the most well-characterized environmental exposures in relation to the placenta. However, there are several opportunities for future directions, including the use of mediation analysis to link exposure and outcome, multi-omics studies with sequencing and methylation data, metal mixture exposures, sex-specific effects/analyses, and expression of non-coding RNAs.

### Air Pollution

Air pollution arises from wildfires, combustion and offgassing-related emissions, manmade energy use and production, and transportation, and it is frequently characterized based on particle size or content. PM_2.5_, also referred to as fine particulate matter, consists of particles less than 2.5 μm in diameter that consist of mixed pollutants. Gestational PM_2.5_ exposures (*N* = 499) and placental gene expression (*N* = 149) were assessed in relation to fetal growth characteristics in the RICHS cohort [22]. PM_2.5_ exposure was positively associated (*p* < 0.05) with gene modules involved in amino acid transport, cellular respiration, and cell adhesion modules and negatively associated with gene modules involved in vasculature and organ development [22]. Two of these gene modules, amino acid transport and cellular respiration, were also associated with infant birth weight percentiles, but with opposite correlation from PM_2.5_ associations [22]. Zhao et al. identified associations between DNA methylation, PM_2.5_ exposure, and fetal and infant growth characteristics in the Shanghai Maternal Child Pairs cohort (*N* = 287) [23]. In this study, 2,098 differentially methylated CpG sites (FDR adjusted *p* < 0.2) were associated with PM_2.5_, including 706 CpGs within gene promoter regions enriched for pathways involving DNA transcription, embryonic organ growth, lipid metabolism, metabolic regulation, and immune responses [23]. Associations between gestational PM_2.5_ exposures in relation to placental DNA methylation, umbilical cord blood vitamin D concentrations, and atopic dermatitis diagnosis between birth and 3 years were evaluated in the Cohort for Childhood Origin of Asthma and Allergic Diseases (COCOA) study (*N* = 922) [24]. In the COCOA study, 195 CpG sites were differentially methylated in relationship with PM_2.5_, vitamin D, and atopic dermatitis [24]. In the EDEN cohort, PM_10_, NO_2_, and humidity were studied as exposures related to DNA methylation (*N* = 668) [25]. Methylation of 4 CpGs (FDR < 0.05) was associated with humidity, nitrogen dioxide (NO_2_), or particulate matter less than 10 μm (PM_10_) [25]. NO_2_ and ozone (O_3_) exposures were compared to placental DNA methylation in the Early Autism Risk Longitudinal Investigation (EARLI) cohort (*N* = 133) [26]. In EARLI, two differentially methylated regions (DMRs) (FWER < 0.05) in the placenta were associated with prenatal NO_2_ exposure [26]. Sex stratified analysis revealed one female specific placental DMR negatively associated with prenatal NO_2_, and two male specific placental DMRs associated with NO_2_ or O_3_ prenatal exposure [26]. Overall, air pollution, especially PM_2.5_, is well-studied; however, there are still several knowledge gaps to fill including transcriptomic analysis of coding and non-coding transcripts, multi-omics studies, and analyses that connect exposures and birth outcomes.

### Psychosocial Stressors

The developing fetus is influenced by multiple aspects of the maternal environment, including intrinsic maternal exposures leading to psychological distress. A wide range of maternal stressors, including maternal mental health, as well as maternal social exposures such as socioeconomic position have been linked to infant and childhood health [55]. In a subset of 581 individuals from the RICHS cohort, 21 imprinted DEGs were associated with maternal depression and anxiety, and 5 imprinted DEGs associated with depression alone, with no differential gene expression in the anxiety only group [44]. Differential methylation at 2833 CpG sites (FDR < 0.05) was associated with maternal depression at 14 weeks in the FinnBrain Birth Cohort Study (*N* = 92) [45]. Genes closest to these CpG sites were enriched in pathways involving generation and development of neurons [45]. Across these two studies of maternal depression, the gene *Erlin2* was identified as a differentially expressed imprinted gene by Litzky et al. and as being adjacent to four differentially methylated CpG sites by Lund et al. [44, 45]. Although a placental specific role for this gene is unknown, its protein is known to be localized to the endoplasmic reticulum where it has functions in protein degradation and lipid metabolism [56]. Maternal socioeconomic position (SEP) has been associated with placental methylation of 33 CpGs (FDR < 0.1) in 426 participants from the Extremely Low Gestational Age Newborns (ELGAN) study [47]. This study identified 15 CpGs associated with marital status, 2 CpGs associated with supplemental nutrition assistance, and one CpG associated with health insurance status [47]. An overall summary score for SEP was associated with 15 overall CpGs, as well as 27 CpGs uniquely in female placentas and 2 CpGs uniquely in male placentas [47]. Placental methylation of 298 CpG sites was associated with maternal night-shift work (FDR < 0.1) in the RICHS cohort (*N* = 237), and these CpGs were located within genes that were involved in cell adhesion [46]. Fourteen of these CpG sites were also associated with altered gene expression in 18 genes (*p* < 0.05) [46].

Psychosocial stress is a category of exposures that is often not considered when evaluating environmental influences on health, and the impact of the psychosocial environment on placental epigenomics remains unclear. Evaluating maternal race as a social construct and component of psychosocial stress is one area of this research in particular that needs more attention. Race is frequently used as a covariate or confounding variable in models assessing epigenomic outcomes in human studies but is rarely treated as a social exposure despite differences in lived experiences across races due to increased risk of environmental exposures, experiences of racism, and reduced opportunities [57, 58]. Future studies should not only evaluate placental epigenomics and infant health outcomes in the context of race as a social construct but also recruit cohorts that accurately model the racial diversity of the general population. Consideration of maternal psychosocial stressors as a component of the gestational environment may yield novel insight into the DOHaD premise. Components of maternal psychosocial stress that may be relevant to future studies have been previously reviewed by Barrero-Castillero et al. [59].

## Placental Epigenomics and Birth Outcomes

Sixteen of the 28 exposure articles reviewed above used placental omics data to link prenatal exposures and accompanying measurements of birth or early childhood health outcomes within the same sample population (Table 1). Methodologies used to connect exposure and outcome results included (1) statistical mediation analyses in a subset of genes, (2) network analyses that identified commonly enriched placental gene networks between exposure and outcome using approaches like weighted gene co-expression network analyses (i.e., WGCNA), or (3) identification of gene or CpG overlaps that were independently associated with the exposure or outcome. A recent study by Clark et al. utilized a novel methodology to connect birth outcomes and environmental exposures through use of a predictive toxicology framework in the ELGAN cohort (*N* = 378) [40]. This study conducted multi-omics analysis of the placenta through measurement of mRNA expression, micoRNA expression, and DNA methylation to identify signatures that may be predictive of perinatal health outcomes, including birth weight, placental weight, placental damage, and placental inflammation [40]. The predictivity of top ranking sites (257 microRNAs) was tested in an independent cohort (*N* = 36) by microarray, identifying 32 overlapping micoRNAs [40]. Of these 32 microRNAs, 6 were found to be associated with prenatal Cd exposure, implying utility of these microRNAs as predictors of perinatal outcomes following prental exposures [40].

Despite the limited number of epigenomic studies integrating both exposures and outcomes, there is a well-established research base of using omics approaches to investigate parturition and birth outcomes. Within the last 5 years, a number of studies have utilized placental epigenomics to gain biological insight into perturbations of the placenta related to birth outcomes including preterm birth, intrauterine growth restriction, birth weight, or birth size, which are summarized in Table 2. This large body of research associating birth outcomes with placental epigenomic measures will be instrumental in developing new hypotheses for exposure-outcome pairings to evaluate through placental epigenomics. For example, Tekola-Ayele et al. [60] evaluated DNA methylation in the NICHD-FGS singleton cohort (*N* = 301) in relation to birth weight. To contexualize the relevance of their findings, pathway analysis was performed on the genes annotated to the top 100 CpG sites associated with birth weight [60]. This analysis identified seven pathways including assembly of RNA polymerase I complex, CTLA4 signaling in cytotoxic T lymphocytes, and DNA methylation and transcriptional repression signaling, among others [60]. Future research can leverage these findings to develop hypotheses surrounding exposure-birth weight associations by identifying exposures that have previously been associated with these same pathways based on placental epigenomics.

**Table 2:** Placental omics birth outcome studies without environmental exposures

Outcome	Omics	Cohort	*N*	Reference
Birth weight	DNA methylation	-	86	Rumbajan 2016 [61]
		NICHD-FGS	301	Tekola-Ayele 2019 [62]
			301	Tekola-Ayele 2020 [60]
	mRNA expression	ELGAN	390	Payton 2020 [63]
	mRNA expression, miRNA expression, DNA methylation	ELGAN	378	Clark 2021 [40]
Fetal growth	DNA methylation	NICHD-FGS	301	Tekola-Ayele 2019 [62]
		-	17	Chen 2018 [64]
			12	Shen 2020 [65]
			51	Díaz 2017 [66]
			154	Rondinone 2021 [67]
	mRNA expression		20	Gibbs 2019 [68]
		RICHS	200	Deyssenroth 2017 [69]
	lncRNA expression, mRNA expression	-	8	Song 2018 [70]
	microRNA expression		31	Thamotharan 2017 [71]
			68	Östling 2019 [72]
Growth restriction	mRNA expression	-	14	Maulik 2016 [73]
			20	Gibbs 2021 [68]
	mRNA expression, H3K27ac		9	Paauw 2018 [74]
	DNA methylation		38	He 2016 [75]
			16	Roifman 2016 [76]
			16	Lee 2021 [77]
			17	Chen 2018 [64]
	circRNA expression		6	Wang 2020 [78]
	microRNA expression		36	Li 2019 [79]
			31	Thamotharan 2017 [71]
Preterm birth	mRNA expression	-	15	Ackerman 2016 [80]
			32	Brockway 2019 [81]
			31	Lien 2021 [82]
			133	Paquette 2018 [83]
		CBCNPBP	90	Wang 2021 [84]
	DNA methylation	-	44	Konwar 2018 [85]
			48	Wang 2019 [86]
			9	Schuster 2019 [87]
			40	Wang 2020 [88]
Autism diagnosis	DNA methylation, mRNA expression	ELGAN	379	Santos 2020 [89]
	DNA methylation	-	20	Bahado-Singh 2021 [90]
Childhood adiposity	DNA methylation	Gen3G	187	Gagné-Ouellet 2020 [91]
Newborn neurobehavior	DNA methylation	RICHS	335	Paquette 2016 [92]

## Limitations and Research Gaps

Sampling of human placentas for use in epidemiological studies poses several challenges. To reduce variability across studies, the majority of epidemiological omics studies sample fetal placental tissue cleared of maternal decidua. Few studies consider the complete maternal–fetal interface. Within each sampling site, the placenta is a heterogenous mix consisting of varied cell populations including cytotrophoblasts, syncytiotrophoblasts, stromal cells, and extravillous trophoblasts. This is a challenge given the cell-type and tissue specificity of most epigenomic markers like DNA methylation, miRNA expression, and histone modifications. Several recent studies have evaluated single cell gene expression in the placenta in a small number of samples to better understand the unique components that make up the bulk tissue [93–95]. Analysis of single cell or specific cell populations of placental tissue is more costly and time-consuming than bulk tissue analysis as it requires fresh rather than frozen samples and thus may not be amenable to research questions that require a large number of samples. Additionally, the timing of placental sampling in comparison to the assessment of exposure or postnatal outcomes varies across studies. The placenta adapts and develops throughout gestation to respond to the changing needs of the developing fetus but is largely only assessed at the end of gestation [96]. Studies involving prenatal exposures thus compare exposure metrics captured during pregnancy with placental omics data at the end of gestation, which may or may not be a direct result of earlier gestational exposures. Maternal and fetal genetics will also play a role in the effect of prenatal exposures on the placenta and developing fetus through gene-environment interactions; however, most studies do not have an adequate sample size to include genetic variation across the complete genome as part of their analysis. Analysis of underlying genetics for select candidate genes identified through an initial assessment of an environmental exposure could allow for incorporation of maternal or fetal genetics into future studies in the context of DOHaD.

There are many opportunities for placental omics data to expand the DOHaD premise. This review concentrated on studies that utilized epigenomic or transcriptomic technology to assess changes in placental gene expression; however, there are many other epigenetic measures that are currently underrepresented in omics studies, including transcriptomic modifiers (such as lncRNAs and microRNAs) or genomic modifiers, such as hydroxymethylation or histone acetylation. Placental exosomes, a type of extracellular vesicle released by the placenta into maternal circulation during pregnancy, are another area of emerging interest. These exosomes secrete microRNAs into maternal circulation, acting as signals elsewhere in the mother or fetus [97, 98], and can be investigated through exosome-specific microRNA sequencing. Utilizing a single study population for assessing multiple epigenomic outcomes in a “multi-omic” approach is another area of interest across all exposure categories. Integration of multiple omics datasets has the advantage of evaluating the combinatorial effects of multiple epigenetic regulators on the outcome of gene expression. Integration of results across currently available studies is limited due to differences in omics platform selection, study design, analysis methods, and availability of full results, thus underscoring the importance of multi-omics studies from within a single study population. Lastly, understanding the effects of RNA editing proteins on the transcriptome and more comprehensive detection of post-transcriptional edits or alternative RNA splicing through deep transcriptome sequencing is another area for further application toward advancement of the DOHaD premise [99].

Connecting gestational exposures and placental epigenomic measures with childhood and later life health outcomes is another area for further research, particularly within cohorts that have follow-up information on children as they enter adolescence from which placental omics data was generated. Although the placenta is an organ of pregnancy that is most commonly associated with birth and early childhood health outcomes, the placenta may still capture developmental changes that extend throughout the lifespan [100]. Identifying placental changes that are associated with risks for diseases in later life would give powerful support to the placenta as a mediating organ of the DOHaD hypothesis.

## Conclusions

In summary, placental DNA methylation and RNA expression have been linked to a broad variety of prenatal exposures that apply widely to human populations. Continued research efforts in placental epigenomics should expand to include analysis of exposure mixtures, produce integrated multi-omic datasets, and connect exposures and outcomes via placental omics measures. The growth of placental epigenomics studies to evaluate the DOHaD hypothesis is promising in its ability to elucidate mechanisms underlying prenatal exposures and health outcomes.
